# Disseminated Aspergillosis with Central Nervous System Involvement Among Patients with Hematologic Malignancies: A 30-Year Institutional Cohort of Clinical Features, Management, and Outcomes

**DOI:** 10.3390/jof12090667

**Published:** 2026-09-04

**Authors:** Saliba Wehbe, Ray Hachem, Anne-Marie Chaftari, Amir Melek, Joanne Arvelaez Pascucci, Ying Jiang, Issam Raad

**Affiliations:** Department of Infectious Diseases, Infection Control and Employee Health, The University of Texas MD Anderson Cancer Center, Houston, TX 77030, USA; salibawehbe@gmail.com (S.W.); rhachem@mdanderson.org (R.H.); melek.amir@gmail.com (A.M.); jarvelaez@mdanderson.org (J.A.P.); yijiang@mdanderson.org (Y.J.); iraad@mdanderson.org (I.R.)

**Keywords:** aspergillosis, CNS, outcomes, cancer patients

## Abstract

**Background:** Disseminated invasive aspergillosis (IA) is an uncommon but devastating manifestation of disease, particularly when the central nervous system (CNS) is involved. The clinical features and outcomes of CNS dissemination, compared with isolated invasive pulmonary aspergillosis (IPA) and other disseminated forms, remain poorly characterized. **Methods:** Using an institutional database of 1157 cancer patients diagnosed with IA between 1993 and 2024, we identified 43 patients with disseminated IA, defined as infection involving ≥2 non-contiguous organ systems, including 8 with CNS involvement. Patients with disseminated IA were matched 1:3 to patients with IPA based on year of diagnosis. We compared clinical features, antifungal treatment, and outcomes between: (1) CNS-disseminated IA and IPA, and (2) CNS-disseminated IA and other forms of disseminated IA. **Results:** Baseline characteristics, including hematologic malignancy subtype, hematopoietic stem cell transplantation status, neutropenia, and antifungal prophylaxis, were similar across groups. *Aspergillus fumigatus* was the predominant species in all cohorts. No patients with CNS-disseminated IA achieved clinical response at end of therapy, versus 35% with IPA (*p* = 0.05) and 30% with other disseminated IA (*p* = 0.17). Twelve-week IA-associated mortality was significantly higher in CNS-disseminated IA than in IPA (88% vs. 45%, *p* = 0.028) and was higher than in other disseminated IA (88% vs. 46%, *p* = 0.05). Combination antifungal therapy was more frequently used in CNS-disseminated IA than in IPA (63% vs. 31%) and other disseminated IA (63% vs. 43%), while rates of ICU admission and mechanical ventilation were similar across groups. **Conclusions:** CNS involvement in disseminated IA defines a distinct, high-risk phenotype, with profoundly reduced treatment response and survival despite comparable baseline characteristics. Given the small number of CNS cases and the predominance of cases diagnosed during earlier study periods, these findings should be interpreted with caution. Although combination antifungal therapy was used more frequently in CNS-disseminated IA, no significant outcome benefit was observed, underscoring the need for improved therapeutic approaches for CNS aspergillosis in immunocompromised hosts.

## 1. Introduction

Invasive aspergillosis (IA) continues to be a prevalent invasive mold infection among patients with hematologic malignancies and recipients of hematopoietic stem cell transplants (HSCTs), carrying substantial morbidity and mortality despite advances in antifungal therapy and supportive care [1,2]. The overall case-fatality rate for IA has declined over recent decades to less than 30% in contemporary cohorts, owing largely to the introduction of mold-active triazoles and improvements in diagnostic modalities [2,3]. Nevertheless, specific anatomic forms of IA continue to be associated with disproportionately severe outcomes, with disseminated and central nervous system (CNS)-involving disease historically associated with mortality rates approaching 100% [1,4].

CNS-involving aspergillosis arises most commonly through hematogenous dissemination from a primary pulmonary focus in profoundly immunosuppressed hosts and manifests as brain abscesses, cerebritis, meningitis, or cerebrovascular complications secondary to angioinvasion [5]. Prior to the availability of voriconazole, CNS aspergillosis was virtually universally fatal, with a crude mortality rate of 99% documented in Denning’s review of 1223 cases [4]. Schwartz and colleagues subsequently demonstrated that voriconazole, which achieves better CNS penetration than other antifungal agents, improved the response rate to 35% and survival rate to 31% in a landmark retrospective analysis of 81 patients with CNS aspergillosis [6]. However, the survival rate among HSCT recipients in that cohort was only 16% [6].

The rarity of CNS-involving disseminated aspergillosis has precluded large-scale comparative studies characterizing its clinical features and outcomes relative to other forms of IA. Most existing data derive from case reports, small case series, or heterogeneous multicenter registries, limiting the ability to draw robust inferences about risk factors and prognosis within specific patient populations.

In this current study, we addressed these knowledge gaps by leveraging a 30-year institutional database of IA among cancer patients at a comprehensive cancer center. We identified all patients with hematologic malignancies and disseminated aspergillosis, with specific attention to those with CNS involvement, and compared their clinical characteristics, treatment patterns, and outcomes against those of matched patients with invasive pulmonary aspergillosis (IPA) alone; we also compared clinical characteristics, treatment patterns, and outcomes between patients with disseminated aspergillosis with and without CNS involvement. The objectives were to delineate the clinical and epidemiologic profile of CNS-involving disseminated aspergillosis, assess temporal trends in its incidence, and evaluate the impact of infection type on therapeutic response and mortality.

## 2. Methods

### 2.1. Study Design, Setting, and Population

This was a retrospective, matched cohort study conducted at The University of Texas MD Anderson Cancer Center, a high-volume tertiary referral institution for hematologic malignancies. An institutional IA database has been prospectively maintained since July 1993 and includes clinical, microbiologic, and outcome data on 1157 cancer patients diagnosed with proven or probable IA through May 2024. All diagnoses were classified according to the European Organization for Research and Treatment of Cancer/Mycoses Study Group consensus definitions [7,8].

From this database, 43 patients with hematologic malignancies who developed disseminated aspergillosis were identified. Dissemination was defined as involvement of two or more non-contiguous organs with or without concurrent pulmonary disease. Among the 43 patients with disseminated aspergillosis, eight patients had documented CNS involvement (the CNS subgroup). Each of the 43 patients with disseminated disease was matched at a 1:3 ratio to patients with IPA alone from the same institutional IA database (n = 129) on the basis of the year of IA diagnosis (same year or one year before or after). Matched IPA controls were randomly selected from the database using simple random sampling without replacement.

### 2.2. Data Collection and Definitions

Data extracted included demographics (age, sex), type of hematologic malignancy (leukemia, lymphoma, myeloma, myelodysplastic syndromes), HSCT history and type (autologous vs. allogeneic), graft-versus-host disease (GVHD) status, neutropenia at IA diagnosis and its persistence, antifungal prophylaxis regimen (liposomal amphotericin B, azoles, echinocandins), IA diagnostic classification (proven vs. probable), microbiologic data (culture positivity, *Aspergillus* species identification), primary antifungal therapy (monotherapy vs. combination therapy), intensive care unit admission, mechanical ventilation, response at end of therapy, IA-associated mortality at 6 and 12 weeks, and all-cause mortality at 6 and 12 weeks.

Persistent neutropenia was defined as neutropenia present at the time of IA diagnosis with no recovery of the absolute neutrophil count above 500 cells/µL for more than 7 days during the study follow-up period.

CNS aspergillosis was defined according to revised EORTC/MSGERC criteria. CNS manifestations included cerebral abscesses, cerebritis, meningeal disease, and cerebrovascular complications attributable to *Aspergillus* infection, including cerebral infarction, intracranial hemorrhage, vasculitis, and mycotic aneurysms. Diagnosis was based on compatible clinical and neuroimaging findings together with microbiological and/or histopathological evidence. Proven CNS aspergillosis required histopathologic demonstration of septate, acute-angle branching hyphae in CNS tissue or isolation of *Aspergillus* species from a sterile CNS specimen. Probable CNS aspergillosis required an appropriate host factor, compatible CNS imaging findings, and mycological evidence of aspergillosis. In patients for whom CNS tissue sampling was not feasible, CNS aspergillosis was inferred from characteristic brain lesions on MRI in the setting of proven or probable extracranial aspergillosis and absence of a more likely alternative diagnosis.

Response to antifungal therapy at 12 weeks was assessed based on the overall treatment outcome documented in the medical record. Although this assessment took into consideration the patient’s clinical, radiographic, and microbiological course, detailed data on each of these response components were not collected or analyzed separately. Overall response was defined as a favorable treatment outcome at the end of therapy, including complete or partial response.

### 2.3. Statistical Analysis

Categorical variables were compared using the chi-square test or Fisher’s exact test, as appropriate. Continuous variables were compared using the Wilcoxon rank-sum test. Multivariable logistic regression was used to identify independent predictors of response to IA therapy, and multivariable Cox regression was used to identify independent predictors of 12-week all-cause mortality, with both models also evaluating the impact of disseminated aspergillosis. Time period of IA diagnosis was included as a covariate to account for temporal changes in management. Two-sided *p*-values < 0.05 were considered statistically significant. All data analyses were performed using SAS version 9.4 (SAS Institute Inc., Cary, NC, USA).

## 3. Results

### 3.1. Demographics and Clinical Characteristics: Disseminated Aspergillosis vs. IPA Alone

The patients with disseminated disease were significantly younger than the patients with IPA alone (median age, 44 vs. 55 years; *p* = 0.046) (Table 1). The sex distribution was similar in the two groups (disseminated, 51% male; IPA alone, 59% male; *p* = 0.374). In both groups, leukemia was the predominant underlying malignancy (72% vs. 71%), followed by lymphoma (28% vs. 22%; *p* = 0.325). HSCT was numerically more common among patients with disseminated disease, although this difference did not reach statistical significance (*p* = 0.060). (53% vs. 37%; *p* = 0.060). In both groups, the vast majority of transplants were allogeneic (87% vs. 83%).

Neutropenia at IA diagnosis was present in 56% of patients with disseminated disease and 47% of patients with IPA alone; however, this numerical difference was not statistically significant (*p* = 0.310). Approximately half of the patients in each group received antifungal prophylaxis (51% vs. 53%; *p* = 0.860).

The proportion of cases classified as proven was significantly higher in the disseminated disease group (65% vs. 29%; *p* < 0.0001), reflecting the diagnostic yield of histopathology and/or culture from extrapulmonary sites. The distribution of *Aspergillus* species was similar in the two groups, with *A. fumigatus* predominating (56% vs. 43%; *p* = 0.184), followed by *A. flavus* (28% vs. 24%).

### 3.2. CNS-Involving Disseminated Aspergillosis

The eight patients (19% [8/43] of the disseminated-disease cohort) with documented CNS or brain involvement exhibited several features distinct from those of the patients with disseminated disease without CNS involvement and the patient with IPA alone. A striking finding was the temporal clustering of CNS cases in the earliest diagnostic era: 63% of CNS cases were diagnosed during 1993–2003, compared with 34% of other disseminated disease cases (*p* = 0.047; Table 2) and 40% of cases of IPA alone (*p* = 0.061; Table 3). Only one CNS case was diagnosed during 2004–2014, and two were diagnosed during 2015–2024 (Table 2).

Patients with CNS involvement were younger (median age, 39 years vs. 46 years for other disseminated disease and 55 years for IPA alone) (Table 2 and Table 3), although these differences did not reach statistical significance. Leukemia was the underlying malignancy in 63% of CNS cases, and 63% of patients with CNS involvement had undergone HSCT, predominantly allogeneic HSCT (80%) (Table 2). Neutropenia at diagnosis was less common among CNS cases than among cases of other disseminated disease (25% vs. 63%), although the difference did not reach statistical significance (*p* = 0.111); persistent neutropenia was infrequent (13%) (Table 2). Half of CNS patients received antifungal prophylaxis, with azoles being the most common class (50%) (Table 2).

The distribution of proven and probable aspergillosis was similar between patients with CNS-involving and non-CNS disseminated aspergillosis (63% vs. 66% proven, respectively; *p* > 0.99). Although proven aspergillosis was more frequent in patients with CNS aspergillosis than in those with IPA alone (63% vs. 29%), this difference did not reach statistical significance (*p* = 0.058). Fungal cultures were positive in 88% of CNS cases. *A. fumigatus* was the most commonly identified species (57%), followed by *A. flavus* (29%) (Table 2). Neither *A. terreus* nor *A. niger* was isolated from CNS cases, whereas both species were present in the other disseminated disease and IPA groups (Table 2 and Table 3).

### 3.3. Treatment Patterns and Outcomes

Combination antifungal therapy was used more frequently in CNS cases (63%) than in cases of other disseminated disease (43%) and IPA alone (31%) (Table 2 and Table 3). Among the CNS patients who received monotherapy, all (3 of 3) were treated with amphotericin B formulations, whereas among the IPA patients who received monotherapy, 47% (41 of 88) received amphotericin B formulations, 39% (34 of 88) were treated with azoles, and 15% (13 of 88) were treated with echinocandins (Table 3).

Intensive care unit admission was required for 63% of CNS patients, compared with 40% of patients with other disseminated disease and 47% of IPA patients (Table 2 and Table 3).

The most salient finding was the uniformly poor therapeutic response and survival among CNS patients. No CNS patient achieved a successful response at the end of IA therapy, compared with 30% (10 of 33) of patients with other disseminated disease (*p* = 0.165; Table 2) and 35% (45 of 127) of IPA patients (*p* = 0.052; Table 3).

The IA-associated mortality rate at 12 weeks was 88% (7 of 8) for CNS patients, compared with 46% for patients with other disseminated disease (*p* = 0.050; Table 2) and 45% for IPA patients (*p* = 0.028; Table 3). The all-cause mortality rate at 12 weeks was 88% for CNS patients, compared with 51% for patients with other disseminated disease (*p* = 0.111; Table 2) and 59% for IPA patients (*p* = 0.147; Table 3).

### 3.4. Multivariable Analyses

In multivariable logistic regression evaluating predictors of successful response to IA therapy, the most powerful predictor was the time period of IA diagnosis: diagnosis during 2004–2014 (vs. 1993–2003) was independently associated with markedly improved odds of response (adjusted odds ratio [aOR], 10.60; 95% CI, 3.97–28.35; *p* < 0.0001) (Table 4). GVHD (aOR, 0.21; 95% CI, 0.07–0.66; *p* = 0.007) and persistent neutropenia (aOR, 0.23; 95% CI, 0.07–0.68; *p* = 0.008) were independently associated with reduced odds of successful response (Table 4). Multivariable Cox regression for 12-week all-cause mortality confirmed the protective effect of later diagnostic era: diagnosis during 2004–2014 (adjusted hazard ratio [aHR], 0.40; 95% CI, 0.26–0.62; *p* < 0.01) and diagnosis during 2015–2024 (aHR, 0.49; 95% CI, 0.25–0.94; *p* = 0.033) were independently associated with reduced mortality risk (Table 5). Persistent neutropenia independently predicted higher mortality risk (aHR, 2.08; 95% CI, 1.35–3.21; *p* < 0.01) (Table 5).

## 4. Discussion

This study represents one of the largest single-institution analyses of disseminated aspergillosis with CNS involvement in patients with hematologic malignancies. The principal findings, that patients had a 0% response rate to IA therapy and an 88% IA-associated mortality rate at 12 weeks, underscore the devastating outcomes of CNS aspergillosis despite contemporary antifungal therapy and multidisciplinary management. These results extend and contextualize prior observations within a well-characterized oncologic population spanning three decades of evolving therapeutic practice.

The fact that CNS aspergillosis is nearly universally fatal has been recognized for decades. In Denning’s 1996 review of 1223 cases of IA, the crude mortality rate for cerebral aspergillosis was 99% [4]. Lin and colleagues, in their systematic review of cases reported after 1995, found a case-fatality rate of 88.1% among patients with disseminated disease or CNS involvement, with only 10 of 84 such patients surviving at the end of follow-up [1]. These estimates predated the widespread use of voriconazole, which was established as first-line therapy for all forms of IA following publication of the landmark randomized trial by Herbrecht and colleagues in 2002 [9]. The 0% response rate and 88% mortality rate observed in the current series despite the availability of voriconazole and other contemporary antifungal agents likely reflect the convergence of severe underlying immunosuppression, the anatomic sanctuary afforded by the blood–brain barrier, and diagnostic delays characteristic of CNS infections in this population.

### 4.1. Temporal Trends and the Declining Incidence of CNS Disease

A noteworthy observation from our study is the clustering of CNS cases in the earliest diagnostic era (1993–2003), with 63% of cases diagnosed before 2004. This temporal decline likely reflects multiple factors. First, the implementation of effective antifungal prophylaxis, particularly with mold-active azoles such as posaconazole and voriconazole, has become standard practice for high-risk patients with hematologic malignancies and HSCT recipients, potentially preventing the pulmonary infections that serve as the nidus for hematogenous dissemination. Second, the adoption of galactomannan-based screening and high-resolution computed tomography has facilitated earlier diagnosis and treatment of pulmonary aspergillosis, which may intercept the disease before dissemination occurs [10]. Third, the widespread use of voriconazole, with its superior CNS penetration, for primary therapy may reduce the probability of cerebral seeding even among patients with established IPA [5].

These improvements are mirrored in the multivariable analyses, which identified diagnosis during 2004–2014 as the most powerful predictor of both therapeutic success (aOR, 10.60; *p* < 0.01) and survival (aHR, 0.40 for 12-week mortality; *p* < 0.01), independent of infection type, GVHD status, and persistent neutropenia. This finding is consistent with broader trends in IA outcomes reported internationally. Pagano and colleagues documented a downward trend in IA-attributable mortality among patients with acute myeloid leukemia in the Italian SEIFEM registry, attributing improvements to the availability of new antifungal agents [3]. Similarly, Kontoyiannis and colleagues and Baddley and colleagues, through the Transplant Associated Infection Surveillance Network, demonstrated declining IA-associated mortality among HSCT recipients, with 12-week mortality rates in the 2000s substantially improved compared with rates from the 1990s [2,11]. However, because most CNS cases in our cohort were diagnosed during the earliest study period, it is difficult to fully distinguish the impact of CNS involvement from the effects of temporal improvements in diagnostic approaches, antifungal therapy, and supportive care.

### 4.2. Unique Characteristics of Patients with CNS-Involving Disseminated Disease

Several features of the patients with CNS involvement merit specific discussion. The relative infrequency of neutropenia at diagnosis (25%) in this group compared with the other disseminated disease (63%) and IPA alone (47%) groups is intriguing, although these differences did not reach statistical significance, and should be interpreted cautiously. This finding may reflect the predominance in the CNS cohort of HSCT recipients (5 of 8 patients), in whom aspergillosis often occurs in the post-engraftment period during immunosuppression for GVHD rather than during the neutropenic phase. Indeed, GVHD was present in 25% of CNS cases. The pathophysiology of CNS involvement in patients without neutropenia may involve qualitative defects in cellular immunity, particularly T-cell-mediated and macrophage-mediated defense mechanisms that are critical for preventing hematogenous dissemination and controlling *Aspergillus* within the CNS parenchyma.

The predominance of *A. fumigatus* (57%) followed by *A. flavus* (29%) in CNS cases is consistent with the published literature on CNS aspergillosis [5,6]. The fact that neither *A. terreus* nor *A. niger* was recovered from CNS cases, though both species were present in the broader disseminated and IPA cohorts, is notable. Whether this finding reflects the differential neurotropism of specific *Aspergillus* species or simply reflects small sample sizes warrants further investigation.

The frequent use of combination antifungal therapy in CNS cases (63%) reflects clinical recognition of the severity of this condition and the limitations of monotherapy. The 2016 Infectious Diseases Society of America guidelines recommend voriconazole as first-line therapy for all forms of IA, with a weak recommendation for consideration of voriconazole plus an echinocandin in selected patients [10]. Although a randomized trial by Marr and colleagues demonstrated a trend toward improved survival with voriconazole plus anidulafungin versus voriconazole alone in the galactomannan-positive subgroup with hematologic malignancies, the overall survival benefit was not significant [12]. In patients with CNS disease, the utility of echinocandins is further limited by their large molecular size, which restricts blood–brain barrier penetration, though recent preclinical data suggest that caspofungin may achieve potentially relevant brain tissue concentrations [5].

Isavuconazole, which demonstrated non-inferiority to voriconazole in the SECURE trial for all-cause mortality at day 42 in patients with invasive mold disease, also penetrates the CNS and represents an alternative first-line agent, though data specific to cerebral aspergillosis remain limited [13]. Posaconazole, which demonstrated non-inferiority to voriconazole in a phase 3 trial, may also warrant investigation for CNS disease, though its CNS pharmacokinetic profile is less well characterized than that of voriconazole [14].

### 4.3. Disseminated Aspergillosis as a Composite Category

An important nuance revealed by these data is that the outcome of disseminated aspergillosis, considered as a composite entity, did not differ significantly from the outcome of IPA alone in either univariable or multivariable analyses. The 12-week IA-associated mortality rate was 53% for all disseminated disease versus 45% for IPA alone, and the adjusted hazard ratio for all-cause mortality for disseminated infection versus IPA was 0.93 (95% CI, 0.58–1.48). This observation suggests that the CNS subgroup accounts for the historically observed excess mortality of disseminated disease. After exclusion of CNS cases, the 12-week IA-associated mortality rate of the remaining disseminated-disease cohort was 46%, essentially identical to the rate for IPA alone. This finding has implications for prognostic classification and risk stratification in clinical trials, in which disseminated aspergillosis is often aggregated as a single high-risk category without distinction between CNS-involving and non-CNS-involving disease.

### 4.4. Prognostic Determinants Beyond Infection Type

The identification of era of diagnosis, GVHD, and persistent neutropenia as prognostic factors for therapeutic response and survival, are concordant with the broader IA literature. Persistent neutropenia has been consistently identified as one of the strongest predictors of adverse outcomes, reflecting the inability to mount effective innate immune responses against angioinvasive hyphae [11]. GVHD, through its dual mechanism of immune dysregulation and the requirement for immunosuppressive therapy, similarly impairs host defense [10]. The powerful prognostic effect of diagnostic era likely reflects improvements in antifungal pharmacotherapy (particularly the transition from amphotericin B-based to voriconazole-based regimens), advances in supportive care, and refinements in diagnostic and therapeutic timing [3,9,10].

### 4.5. Limitations

Several limitations of this study should be acknowledged. First, the small sample size of the CNS subgroup (n = 8) substantially limits statistical power and increases the risk of Type II error. Therefore, the absence of statistically significant differences should be interpreted with caution, and the small sample size precludes definitive inference regarding several comparisons. Second, the retrospective design introduces potential biases, including ascertainment bias in the detection of CNS involvement, which may have resulted in disproportionate identification of more severe or clinically apparent cases. In addition, CNS involvement may have been underestimated because of the well-recognized diagnostic challenges of CNS aspergillosis and the possibility that occult disease remained undetected. Third, the 30-year study span encompasses substantial shifts in diagnostic criteria, antifungal treatment, and transplant practices, creating inherent heterogeneity within the cohort that complicates interpretation. Additionally, most CNS cases occurred during earlier study periods, when diagnostic capabilities and therapeutic options were more limited; therefore, some of the observed differences in outcomes may reflect temporal changes in management rather than the effect of CNS involvement alone. Fourth, as this is a single-institution study at a large cancer center, the findings may not be generalizable to non-oncologic or community settings. An additional limitation is that detailed data on the status of the underlying hematologic malignancy (e.g., remission, relapsed, or refractory disease) were not consistently available because of the retrospective design and the extended study period. Consequently, we were unable to assess the impact of disease status on outcomes (treatment response and survival). Persistent neutropenia may have served as a partial surrogate for ongoing immunosuppression and/or uncontrolled disease; however, this relationship could not be formally evaluated in the present study. Future studies incorporating detailed malignancy status may provide additional insight into factors influencing treatment response and survival. Additionally, detailed data on neurosurgical interventions, which Schwartz and colleagues identified as an independent predictor of improved survival in CNS aspergillosis [6], were not available for the present analysis. Finally, because of the retrospective nature of the study, follow-up beyond 12 weeks was unavailable, precluding assessment of 6-month mortality and other longer-term outcomes.

## 5. Conclusions

This study, the largest single-institution analysis of CNS-involving disseminated aspergillosis in patients with hematologic malignancies, demonstrated a 0% therapeutic response rate and 88% IA-associated mortality rate at 12 weeks. Outcomes were significantly worse than those observed in IPA alone and numerically worse than those observed in other forms of disseminated disease. However, comparisons between CNS and non-CNS disseminated disease should be interpreted cautiously given the small number of CNS cases. While the overall incidence of CNS aspergillosis appears to be declining, likely a reflection of improvements in antifungal prophylaxis and therapy, outcomes in patients with established CNS disease remain poor. Because most CNS cases occurred during earlier study periods, the contribution of temporal changes in diagnostics and management to these outcomes cannot be fully excluded. Given the small number of CNS cases, treatment-related findings should be interpreted with caution; however, these results highlight the ongoing challenges in managing CNS aspergillosis. Our findings underscore the need for heightened clinical vigilance in high-risk populations, development of rapid CNS-directed diagnostics, and continued investigation of novel antifungal agents and adjunctive therapies. They also suggest that separating CNS-involving from non-CNS-involving disseminated aspergillosis may be valuable in prognostic models and future clinical trial design.

## Figures and Tables

**Table 1:** Comparison of Disseminated Aspergillosis and IPA Alone among Patients with Hematologic Malignancies.

Variable	Disseminated Aspergillosis	IPA Alone	*p*-Value
	(n = 43)	(n = 129)	
	N (%)	N (%)	
Time period of *Aspergillus* diagnosis ^a^			>0.999
1993–2003	17 (40)	51 (40)	
2004–2014	21 (49)	63 (49)	
2015–2024	5 (12)	15 (12)	
Age, median (range), y	44 (9–80)	55 (12–85)	0.046
Sex			0.374
Male	22 (51)	76 (59)	
Female	21 (49)	53 (41)	
Type of hematologic malignancy			0.325
Leukemia	31 (72)	92 (71)	
Lymphoma	12 (28)	28 (22)	
Myeloma	0 (0)	8 (6)	
Myelodysplastic syndrome	0 (0)	1 (1)	
HSCT	23 (53)	48 (37)	0.060
Type of HSCT			>0.999
Autologous	3/23 (13)	8/47 (17)	
Allogeneic	20/23 (87)	39/47 (83)	
GVHD	11 (26)	23/127 (18)	0.290
Neutropenia at diagnosis	24 (56)	60/128 (47)	0.310
Persistent neutropenia ^b^	8/42 (19)	36/126 (29)	0.224
Prophylaxis against *Aspergillus*	22 (51)	68 (53)	0.860
Amphotericin B	5 (12)	18 (14)	0.698
Azole	17 (40)	44 (34)	0.520
Echinocandin	4 (9)	11 (9)	>0.999
Aspergillosis classification			<0.0001
Proven	28 (65)	37 (29)	
Probable	15 (35)	92 (71)	
Positive fungal culture	40 (93)	121 (94)	>0.999
*Aspergillus* species ^c,d^			
*A. fumigatus*	20/36 (56)	48/112 (43)	0.184
*A. flavus*	10/36 (28)	27/112 (24)	0.658
*A. terreus*	4/36 (11)	21/112 (19)	0.287
*A. niger*	4/36 (11)	10/112 (9)	0.745
Other	3/36 (8)	11/112 (10)	>0.999
Primary antifungal therapy			>0.999
No therapy	1 (2)	1 (1)	
Monotherapy	22 (51)	88 (68)	
Combination therapy	20 (47)	40 (31)	
Type of primary monotherapy			
Amphotericin B	14/22 (64)	41/88 (47)	0.153
Azole	6/22 (27)	34/88 (39)	0.322
Echinocandin	2/22 (9)	13/88 (15)	0.731
ICU admission	19 (44)	60 (47)	0.791
Mechanical ventilation	13 (30)	45 (35)	0.576
Response at end of therapy	10/41 (24)	45/127 (35)	0.190
IA-associated mortality			
At 6 weeks	16 (37)	49/128 (38)	0.900
At 12 weeks	23 (53)	57/126 (45)	0.350
All-cause mortality			
At 6 weeks	18 (42)	63 (49)	0.427
At 12 weeks	25 (58)	76 (59)	0.929

IPA, invasive pulmonary aspergillosis. ^a^ Percentages may not sum to exactly 100% due to rounding. ^b^ Persistent neutropenia refers to neutropenia present at IA diagnosis with no recovery for more than 7 days during the study follow-up. ^c^ *Aspergillus* species were not identifiable in some patients with a positive *Aspergillus* culture. ^d^ Some patients had more than one *Aspergillus* species identified in their positive culture.

**Table 2:** Comparison of CNS-Involving and Other Disseminated Aspergillosis in Patients with Hematologic Malignancies.

Variable	CNS-Involving Disseminated Aspergillosis	Other Disseminated Aspergillosis	*p*-Value
	(n = 8)	(n = 35)	
	N (%)	N (%)	
Time period of *Aspergillus* diagnosis ^a^			0.047
1993–2003	5 (63)	12 (34)	
2004–2014	1 (13)	20 (57)	
2015–2024	2 (25)	3 (9)	
Age (years), median (range)	39 (23–77)	46 (9–80)	0.574
Gender			0.457
Male	3 (38)	19 (54)	
Female	5 (63)	16 (46)	
Type of hematological malignancy			0.665
Leukemia	5 (63)	26 (74)	
Lymphoma	3 (38)	9 (26)	
HSCT	5 (63)	18 (51)	0.704
Type of HSCT			0.539
AUTO	1/5 (20)	2/18 (11)	
ALLO	4/5 (80)	16/18 (89)	
GVHD	2 (25)	9 (26)	>0.99
Neutropenia at diagnosis	2 (25)	22 (63)	0.111
Persistent neutropenia ^b^	1 (13)	7 (21)	>0.99
prophylaxis against *Aspergillus*	4 (50)	17 (49)	>0.99
Amphotericin B	1 (13)	4 (11)	>0.99
Azole ^c^	4 (50)	13 (37)	0.692
Voriconazole	0 (0)	6 (17)	0.574
Posaconazole	2 (25)	3 (9)	0.228
Isavuconazole	0 (0)	0 (0)	
Itraconazole	2 (25)	5 (14)	0.597
Echinocandin	0 (0)	4 (11)	>0.99
Aspergillosis classification			>0.99
Proven	5 (63)	23 (66)	
Probable	3 (38)	12 (34)	
Positive fungal culture	7 (88)	33 (94)	0.470
*Aspergillus* species ^d,e^			
*A. fumigatus*	4/7 (57)	16/29 (55)	>0.99
*A. flavus*	2/7 (29)	8/29 (28)	>0.99
*A. terreus*	0/7 (0)	4/29 (14)	0.566
*A. niger*	0/7 (0)	4/29 (14)	0.566
Other	1/7 (14)	2/29 (7)	0.488
Primary antifungal therapy			0.549
No therapy	0 (0)	1 (3)	
Monotherapy	3 (38)	19 (54)	
Combination therapy	5 (63)	15 (43)	
Type of primary monotherapy			
Amphotericin B	3/3 (100)	11/19 (58)	0.273
Azole	0/3 (0)	6/19 (32)	0.533
Echinocandin	0/3 (0)	2/19 (11)	>0.99
ICU	5 (63)	14 (40)	0.432
Mechanical ventilation	2 (25)	11 (31)	>0.99
Response at end of therapy	0 (0)	10/33 (30)	0.165
IA-associated mortality			
At 6 weeks	4 (50)	12 (34)	0.443
At 12 weeks	7 (88)	16 (46)	0.050
All-cause mortality			
At 6 weeks	4 (50)	14 (40)	0.701
At 12 weeks	7 (88)	18 (51)	0.111

^a^ Percentages may not sum to exactly 100% due to rounding. ^b^ Persistent neutropenia refers to neutropenia present at IA diagnosis with no recovery for more than 7 days during the study follow-up. ^c^ One patient received more than one anti-mold azole for antifungal prophylaxis. ^d^ *Aspergillus* species were not identifiable in some patients with a positive *Aspergillus* culture. ^e^ Some patients had more than one *Aspergillus* species identified in their positive culture.

**Table 3:** Comparison of CNS-Involving Disseminated Aspergillosis and IPA Alone in Patients with Hematologic Malignancies.

Variable	CNS-Involving Disseminated Aspergillosis	IPA Alone	*p*-Value
	(n = 8)	(n = 129)	
	N (%)	N (%)	
Time period of *Aspergillus* diagnosis ^a^			0.061
1993–2003	5 (63)	51 (40)	
2004–2014	1 (13)	63 (49)	
2015–2024	2 (25)	15 (12)	
Age (years), median (range)	39 (23–77)	55 (12–85)	0.138
Gender			0.283
Male	3 (38)	76 (59)	
Female	5 (63)	53 (41)	
Type of hematological malignancy			0.657
Leukemia	5 (63)	92 (71)	
Lymphoma	3 (38)	28 (22)	
Myeloma	0 (0)	8 (6)	
Myelodysplastic syndromes (MDS)	0 (0)	1 (1)	
HSCT	5 (63)	48 (37)	0.261
Type of HSCT			>0.99
AUTO	1/5 (20)	8/47 (17)	
ALLO	4/5 (80)	39/47 (83)	
GVHD	2 (25)	23/127 (18)	0.641
Neutropenia at diagnosis	2 (25)	60/128 (47)	0.290
Persistent neutropenia ^b^	1 (13)	36/126 (29)	0.444
prophylaxis against *Aspergillus*	4 (50)	68 (53)	>0.99
Amphotericin B	1 (13)	18 (14)	>0.99
Azole	4 (50)	44 (34)	0.451
Voriconazole	0 (0)	16 (12)	0.596
Posaconazole	2 (25)	8 (6)	0.106
Isavuconazole	0 (0)	3 (2)	>0.99
Itraconazole	2 (25)	17 (13)	0.307
Echinocandin	0 (0)	11 (9)	>0.99
Aspergillosis classification			0.058
Proven	5 (63)	37 (29)	
Probable	3 (38)	92 (71)	
Positive fungal culture	7 (88)	121 (94)	0.428
*Aspergillus* species ^c,d^			
*A. fumigatus*	4/7 (57)	48/112 (43)	0.698
*A. flavus*	2/7 (29)	27/112 (24)	0.678
*A. terreus*	0/7 (0)	21/112 (19)	0.351
*A. niger*	0/7 (0)	10/112 (9)	>0.99
Other	1/7 (14)	11/112 (10)	0.535
Primary antifungal therapy			0.167
No therapy	0 (0)	1 (1)	
Monotherapy	3 (38)	88 (68)	
Combination therapy	5 (63)	40 (31)	
Type of primary monotherapy			
Amphotericin B	3/3 (100)	41/88 (47)	0.109
Azole	0/3 (0)	34/88 (39)	0.290
Echinocandin	0/3 (0)	13/88 (15)	>0.99
ICU	5 (63)	60 (47)	0.477
Mechanical ventilation	2 (25)	45 (35)	0.715
Response at end of therapy	0 (0)	45/127 (35)	0.052
IA-associated mortality			
At 6 weeks	4 (50)	49/128 (38)	0.711
At 12 weeks	7 (88)	57/126 (45)	0.028
All-cause mortality			
At 6 weeks	4 (50)	63 (49)	>0.99
At 12 weeks	7 (88)	76 (59)	0.147

^a^ Percentages may not sum to exactly 100% due to rounding. ^b^ Persistent neutropenia refers to neutropenia present at IA diagnosis with no recovery for more than 7 days observed during the study follow-up. ^c^ *Aspergillus* species were not identifiable in some patients with a positive *Aspergillus* culture. ^d^ Some patients had more than one *Aspergillus* species identified in their positive culture.

**Table 4:** Multivariable Logistic Regression Analysis of Independent Predictors of Successful Response to IA Therapy and impact of disseminated infection.

Variable	aOR	95% CI	*p*-Value
Time period of IA diagnosis			<0.01
1993–2003	Reference		
2004–2014	10.60	3.97–28.35	<0.01
2015–2024	3.29	0.67–16.19	0.143
GVHD	0.21	0.07–0.66	0.007
Persistent neutropenia ^a^	0.23	0.07–0.68	0.008
Type of IA infection			0.319
Disseminated infection	0.62	0.24–1.59	
Invasive pulmonary infection	Reference		

^a^ Persistent neutropenia refers to neutropenia present at IA diagnosis with no recovery for more than 7 days during the study follow-up. Abbreviations: aOR, adjusted odds ratio; 95% CI, 95% confidence interval.

**Table 5:** Multivariable Cox Regression Analysis of Independent Predictors of 12-Week All-Cause Mortality and impact of disseminated infection.

Variable	aHR	95% CI	*p*-Value
Time period of IA diagnosis			<0.01
1993–2003	Reference		
2004–2014	0.40	0.26–0.62	< 0.01
2015–2024	0.49	0.25–0.94	0.033
Persistent neutropenia ^a^	2.08	1.35–3.21	<0.01
Type of IA infection			0.745
Disseminated infection	0.93	0.58–1.48	
Invasive pulmonary infection	Reference		

^a^ Persistent neutropenia refers to neutropenia present at IA diagnosis with no recovery for more than 7 days during the study follow-up. Abbreviations: aHR, adjusted hazard ratio; 95% CI, 95% confidence interval.

## Data Availability

The data underlying this study are not publicly available due to privacy and ethical restrictions protecting patient confidentiality.
